# Forced duction training: A potential key point for recovery in pediatric patients with trapdoor fracture: Erratum

**DOI:** 10.1097/MD.0000000000006498

**Published:** 2017-03-24

In the article “Forced duction training: A potential key point for recovery in pediatric patients with trapdoor fracture”,^[1]^ which appeared in Volume 95, Issue 44 of *Medicine*, Drs. Yinwei Li and Xuefei Song should have been noted as co-first authors who contributed equally to this article. Also, Drs. Xianqun Fan and Ming Lin should have been reported as co-corresponding authors who contributed equally to the article.
